# Numerical simulations in two-dimensional neural fields

**DOI:** 10.1186/1471-2202-16-S1-P22

**Published:** 2015-12-18

**Authors:** Pedro M Lima, Evelyn Buckwar

**Affiliations:** 1 Institute of Stochastics, Johannes Kepler University, 4040, Linz, Austria; 2 CEMAT/Instituto Superior Técnico, University of Lisbon, 1049, Lisboa, Portugal

## 

We present and discuss a new numerical algorithm for solving the two-dimensional Neural Field Equation with space-dependent delays:

$$\begin{array}{c} c\frac{\partial }{\partial t}V(\bar{x},t)=I(\bar{x},t)-V(\bar{x},t)+\int_{\Omega }K(|\bar{x}-\bar{y}|)S(V(y,t-\tau (\bar{x},\bar{y}))d\bar{y}\\ t\in [0,T],\;\bar{x}\in \Omega \subset {\mathbb{R}}^{2} \end{array}$$

used in Neuroscience to describe the evolution of a population of neurons and the interactions between them. A similar equation (without delays) was first introduced by Wilson and Cowan [1], and then by Amari [2]. Here *V(**x**,t) *represents the post-synaptic membrane potential at instant t and position ***x***. The function I represents external sources of excitation and S describes the dependence between the firing rate of the neurons and their membrane potential (typically it is a function of sigmoidal type). The kernel function *K(|**x-y**|) *describes the connectivity between the neurons at positions × and y. The delay τ(*x,y*) takes into the consideration the time spent by an electrical signal to travel between these two positions.

The new numerical method presented in this work is based on an implicit second order method for discretisation in time and uses Gaussian quadratures for space integration. We use low-rank methods to reduce the computational effort, which enables to reduce very significantly the dimensions of the involved matrices, without affecting the final accuracy of the method.

This algorithm is targeted directly to the application in Neuroscience and Robotics. As an illustration of such applications, we consider a neural field described in [3], where the firing rate function has the form *S(x)= 2/ (1+exp(- μ x)) *(μ is a certain positive constant), and the connectivity function is given by *K(x)= 1/(2 πξ12)1/2 exp(- ||x||2/ 2 ξ12) - A/(2 πξ22)1/2 exp(- ||x||2/ 2 ξ22)*, with *A, ξ1, ξ2 *given positive constants.

It is known [3] that the stability of the trivial solution of this equation depends on the value of μ and on the parameters of the connectivity function. In particular, for each set of values of these parameters, it is possible to compute a bifurcation value μ_bif_, such that if μ < μbif the zero solution is stable, and otherwise it is unstable.

With the help of our algorithm, these bifurcation values can be efficiently computed. Some examples are displayed in Table 1 . The presented values are for the case *τ(x,y)=0*.

**Table 1:** 

A	ξ_1_	μ_bif_	A	ξ_1_	ξ_2_	μ_bif_
0	0.1	15.81	1	0.4	0.3	17.68
0	0.2	7.98	1	0.5	0.3	10.24
0	0.3	5.35	1	0.6	0.3	8.41
0	0.4	4.06	1	0.7	0.3	7.94
0	0.5	3.56	1	0.8	0.3	8.09

## Ackknowledgement

This research was supported by a Marie Curie Intra European Fellowship, PIEF-GA-2013-629496.
